# Male migration and risky sexual behavior in rural India: is the place of origin critical for HIV prevention programs?

**DOI:** 10.1186/1471-2458-11-S6-S6

**Published:** 2011-12-29

**Authors:** Niranjan Saggurti, Bidhubhusan Mahapatra, Suvakanta N Swain, Anrudh K Jain

**Affiliations:** 1 HIV and AIDS Program, Population Council, New Delhi - 110003, India; 2 Distinguished Scholar, Population Council, New York, USA

## Abstract

**Background:**

Recent studies of male migrants in India indicate that those who are infected with HIV are spreading the epidemic from high risk populations in high prevalence areas to populations in low prevalence areas. In this context, migrant men are believed to initiate and have risky sexual behaviors in places of destination and not in places of origin. The paucity of information on men's risky sexual behaviors in places of origin limits the decision to initiate HIV prevention interventions among populations in high out-migration areas in India.

**Methods:**

A cross-sectional behavioral survey was conducted among non-migrants, returned migrants (with a history of migration), and active (current) migrants in rural areas across two districts with high levels of male out-migration: Prakasam district in Andhra Pradesh and Azamgarh district in Uttar Pradesh. Surveys assessed participant demographics, migration status, migration history, and sexual behavior along the migration routes, place of initiation of sex. District-stratified regression models were used to understand the associations between migration and risky sexual behaviors (number of partners, condom use at last sex) and descriptive analyses of migrants' place of sexual initiation and continuation along migration routes.

**Results:**

The average age at migration of our study sample was 19 years. Adjusted regression analyses revealed that active migrants were more likely to engage in sex with sex workers in the past 12 months (Prakasam: 15 percent vs. 8 percent; adjusted odds ratio (aOR)=2.1, 95% CI 1.2-3.4; Azamgarh: 19 percent vs.7 percent; aOR=4.0, 95% CI 2.4-6.6) as well as have multiple (3+) sex partners (Prakasam: 18 percent vs. 9 percent; aOR=2.0, 95% CI 1.3-3.2; Azamgarh: 28 percent vs. 21 percent; aOR=1.9, 95% CI 1.2-3.0) than non-migrants. Contrary to popular belief, a high proportion of active and returned migrants (almost 75 percent of those who had sex) initiated sex at the place of origin before migrating, which is equivalent to the proportion of non-migrants who engaged in sex with sex workers as well as with casual unpaid partners. Moreover, non-migrants were more likely than migrants to engage in unprotected sex.

**Conclusion:**

Findings of this study document that returned migrants and active migrants have higher sexual risk behaviors than the non-migrants. Most migrants initiate non-marital sex in the place of origin and many continue these behaviors in places of destination. Migrants’ destination area behaviors are linked to sex with sex workers and they continue to practice such behaviors in the place of origin as well. Unprotected sex in places of destination with high HIV prevalence settings poses a risk of transmission from high risk population groups to migrants, and in turn to their married and other sexual partners in places of origin. These findings suggest the need for controlling the spread of HIV among both men and women resulting from unsafe sex in places of origin that have high vulnerability due to the frequent migratory nature of populations.

## Introduction

In the context of global industrialization and urbanization, migration for work is increasing around the world [1-3]. Migrant workers seeking employment in urban settings are often from tribal and rural communities with low levels of literacy and skills, making them ignorant about available HIV prevention services resulting in low utilization of these services at the places of destination [2]. A number of studies have documented that migrants have higher risky sexual behaviors than non-migrants [4-12] and that they serve as bridge population for spreading HIV from destination areas to their place of origin [13-16]. Migrants’ sexual relationships with multiple partners in destination areas is assumed to be the main factor explaining the role of migration in the spread of HIV and other STIs [17]. It is widely believed that migrant men acquire infections at the workplace and continue to have sexual contact with their female partners upon returning to their native place, and hence spread infection from destination areas to places of origin [18,19].

Migration within India parallels the global phenomenon [1]. Published research studies on migration in India are limited to the risky sexual behavior of migrants at destination points [1,20-27]. Such research studies in India as well as other parts of the world suggest that migrants initiate and engage in risky sexual behaviors in places of destination due to separation from their family and spouse for extended periods [21,23,26,28,29], isolation coupled with loneliness [26,29], socio-cultural norms and the anonymity of living in a city [21,26,30], illegal residential status [29] and the nature of work [23,26,29]. These studies recommend that the destination areas and the work place are appropriate sites to reach migrant workers with HIV prevention interventions. A recently conducted study in the southern states of India of male migrants at their place of destination found that more than 30 percent had sex with women who were not their married partner in the place of origin [1,22] and about 10 percent had sex with sex workers. These results suggest that sex with sex workers is not limited to the city/town where migrant men work; rather such practices also exist in the place of origin; however, it is not known whether these men initiate such risky behaviors in the place of destination or the place of origin.

Few studies have been conducted to examine the extent of risky sexual behaviors among male migrants in their place of origin in India. A recent study of young unmarried migrants revealed that they were more likely to have had sex at the place of origin - before and after migration - than at the place of destination. The study also reported that 24 percent of migrants had ever had sex at the place of destination, and 80 percent of them reported having sex at the place of origin as well [31]. Despite the recommendations from such studies, places of origin have been neglected in research as well as in HIV prevention programs, may be in part due to the difficulty in identifying such places.

In order to fill the gap in understanding the need and urgency for initiating HIV prevention programs in places of origin, it is important to ascertain where migrant men initiate risky sexual behaviors and how they differ from non-migrants in such places. This area of research is important in view of rising HIV prevalence among antenatal care (ANC) clinic attendees in districts with high male out-migration as well in view of the fact that migrant men studied in destination areas report that they have engaged in risky sexual behavior even in their native places. This study addresses the following research questions to draw implications for initiating HIV prevention programs in places of origin as well: (i) Is sex behavior of migrants different from non-migrants in places of origin?; (ii) Where do migrants initiate commercial sex and sex with casual unpaid partners?; (iii) What proportion of men continue to practice risky sexual behaviors in destination and origin areas, after initiating sex in their place of origin?

## Methods

**Figure 1:**
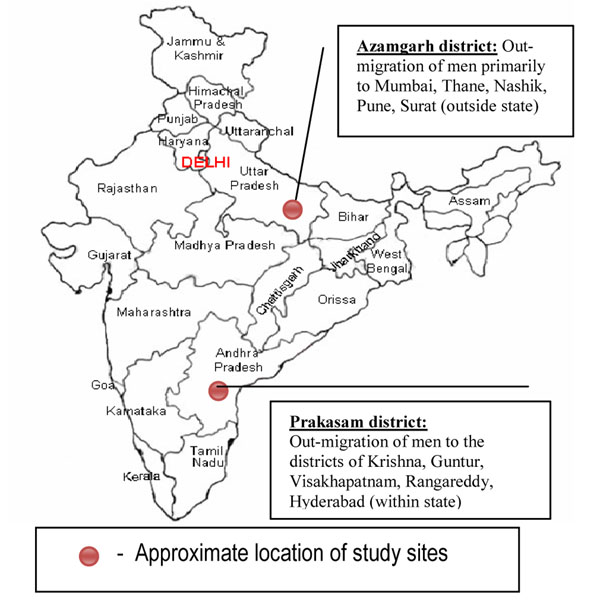
Map showing the approximate geographic locations of study sites within each state in India and the major routes of male out-migration.

This paper reports on research conducted as part of a 5-year (2005-10) knowledge building grant assessing the patterns of male migration and its relationship with HIV risk behaviors from 21 districts with high in-migration across four states in southern (Andhra Pradesh, Karnataka, Tamil Nadu) and western (Maharashtra) India, identified as high HIV epidemic states by the Indian National AIDS Control Organisation (NACO) prior to the year 2005. Districts with the highest rates of in-migration in each state were chosen, as per the 2001 Indian Census. The overall research design of the project has been described in detail elsewhere [1]. The data reported in this study is an extension of the in-migration (or destination) districts study, with a cross-sectional behavioral survey conducted among migrant and non-migrant men in two districts with high levels of out-migration. The two districts for this extended study were chosen based on the places of origin mentioned by migrants in the destination districts study [1,22] with the criterion that one district contains out-migration of men within the state (Prakasam district in Andhra Pradesh) and another district has out-migration of men outside the state (Azamgarh district in Uttar Pradesh) Figure 1).

### Study setting

The study was conducted in Azamgarh district, one of 71 districts in Uttar Pradesh, a state in north India. Azamgarh district has a population of 4.6 million as per the 2011 Census, and is characterized by low female literacy (63 percent) [32], high poverty (57 percent of households belonged to the lowest two categories of wealth quintiles) [33], lack of employment and high levels of out-migration. According to the 2001 Census data on migration, about 15 percent of males in the adult ages have migrated outside the district; and 75 percent of those who had migrated travelled outside the state for work. Data shows that male out-migration is a on a continuous increase since 1980s. The most preferred destination areas for migrants from the district are Mumbai and Thane in Maharashtra. Migrant men from the district work in the unorganized sector including as taxi drivers, daily wage laborers or in the cotton industry as laborers. In the recent HIV sentinel surveillance conducted by NACO, the HIV prevalence among ANC clinic attendees in Azamgarh district is close to 1 percent , which is above the national average of 0.3 percent [34].

Prakasam district, located in coastal Andhra Pradesh in south India, has a population of 3.4 million as per the 2011 Census. The district is spread across 17,626 sq.km, and is characterized by low literacy (63 percent) [35] and 24 percent belong to low economic status [36]. As per the 2001 Census data, 14 percent of male adults had migrated outside the district for work, of which 95 percent had migrated to districts within the state. Migration of males even within the district is common as there are a few industrial and mining establishments within the district. The most preferred destination districts for migrants from Prakasam are: Guntur and Krishna, which are neighboring districts. According to the recent HIV sentinel surveillance, the HIV prevalence among ANC clinic attendees is 2.6 percent which is significantly higher than the state average of 1.4 percent [34].

### Participants

The study included a survey among three categories of men: (i) return migrants, defined as those who had returned to their native place (for at least one year) either due to completion of job contract or no job at the destination place and/or employed locally; (ii) active migrants, defined as those who are temporarily visiting the place of origin (e.g., to attend a marriage or some other function, on vacation, or illness) but are currently employed in a district other than the place of origin; (iii) non-migrants are those who had never moved out of their native place for work.

### Sampling design

The first step in the sampling procedure was to estimate the sample size to detect a difference of at least 7 percent in sexual behavior outside marriage (ever) between non-migrants and returned or active migrants with 80 percent power using a cutoff for statistical significance of 0.05. These proportions indicated a sample of approximately 300 per group; we purposefully inflated the sample size of non-migrants (to cover 400 instead of 300), assuming that risky sexual behaviors would be significantly lower in this group.

The second step in the sampling procedure involved the identification and selection of villages with a sufficient number of out-migrants to attain the desired sample size for each of the three categories in each district. We first selected three *tehsils* (sub-district areas) from each district based on the *tehsil* name mentioned by migrants as their native place in the destination district study [1]. Several key individuals in each *tehsil*—staff from the block development office, primary health centres (PHCs), local non-governmental organizations (NGOs) and officials from revenue offices—were contacted to find out the villages from where most men out-migrate. These key individuals guided the preparation of the list of villages and the approximate percentage of households consisting at least one male member who had worked in the past or was currently working outside the district. Six villages from each *tehsil* were randomly chosen following the preparation of the list of villages.

The third step involved the preparation of household lists and the selection of households. It was decided to fix the number of completed interviews at 50-60 men per village with an approximate target of 40 percent non-migrants, 30 percent returned migrants and 30 percent active migrants. From the household lists in each village, 80 households per village were randomly selected estimating a 20 percent loss. In each selected household, a key individual (usually the head of the household) was asked to give information on himself (or herself), and other family members, and the migration status of the men. From the list of household members, one eligible individual (>18 years old, non-migrant, returned migrant or active migrant) from each household was randomly selected using KISH tables (a method developed by Lesley Kish), which allows the data collection team to randomly select potential participants with equal probability from the list of eligible household members [37]. Through this approach, 1,440 households were contacted from each district, and interviews conducted with men from a total of 2,104 households across two districts: 1,034 from Prakasam district and 1,070 from Azamgarh district. Of the remaining 776 households, in 680 households all the men were working outside the district at the time of survey, in 76 households men refused to participate in the survey, and in 20 households men did not complete the interview and were excluded from the database.

### Ethical considerations

Procedures for this study were reviewed and approved by the institutional review board of the Population Council. Verbal consent was obtained from all respondents before the interview.

### Assessment

Participants received a 45 minute interviewer-administered questionnaire in Hindi (for Azamgarh district) and Telugu (for Prakasam district) assessing demographics, migration history, sex risk behaviors, and history of STI-like symptoms. Survey questionnaires were developed in English and then translated into the local language. The translated questionnaires were reviewed by a study investigator fluent in all three languages. Interviews were conducted by graduates or postgraduates in sociology, anthropology or statistics. Interviewers were experienced in quantitative data collection techniques and field-based public health and HIV/AIDS research. They were trained in data collection using the questionnaire for this study. Data quality and management involved immediate review of the completed questionnaire by each interviewer after completing the interview to ensure accuracy and completeness, same-day review by the supervisor, and weekly transport of questionnaires to the data management team in Delhi. Trained data entry officers entered the data weekly and processed it to verify consistency and accuracy, using SPSS.

### Measures

Demographic data were collected based on questions modified or taken from the Demographic and Health Survey and Population Council surveys and included age, level of education, income, religion, marital status, and number of children.

Sexual behaviors were assessed for each type of sex partners: sex worker, casual unpaid sex partner, and male sex partner. For each type of partner, participants were asked about the number of partners with whom they had sex as well as the number of sexual encounters in their lifetime and in the past year. They were also asked, again by type of partner, the frequency of unprotected sex out of all sex encounters (indicated by condom use in number of sex episodes) in the past year. All these items were used to provide descriptive data on the sexual behaviors of the population.

To determine the place where migrants had initiated such behaviors-- in the place of destination or place of origin-- migrants (both returned as well as active) were asked to indicate by partner type, their history of sex with sex workers at the place of origin prior to first migration, at the places of destination during migration, and the place of origin during past and current visits. Participants were also asked about whether or not they had used condom all the times that they had sex in each of these places (coded as 1=yes, used all the times; 2=no, not used all the times).

### Data analysis

All analyses were conducted separately for Prakasam and Azamgarh districts due to different patterns of male out-migration as well as different cultural norms and behaviors in these two regions. Basic descriptive analyses were run on selected socio-demographic characteristics to describe the sample. All analyses were performed using SPSS software (version 16.0; SPSS Inc.).

### Association between migration status of the participants and risky sexual behaviors

The outcome variables used in the analyses were: (a) ever had sex with sex workers or casual unpaid female partners, (b) ever had sex with a male partner, (c) consistent condom use in sex with female sex workers and casual unpaid partners in the past year, and (d) overall risky sexual behavior (computed from the following indicators: had sex with both sex worker and casual unpaid partners, inconsistent condom use with sex workers and inconsistent condom use with casual unpaid partners in the past year). Migration status of the individual was the main independent variable of interest. Logistic regression models were used to estimate the effects of migration status after controlling for potential demographic confounders such as age, education, marital status (currently married vs. not currently married) and occupation (agriculture vs. others). To avoid possible collinearity, pair-wise Spearman correlations between the independent variables and covariates were assessed prior to regression modelling, and no covariate from the pair of variables with correlation greater than 0.40 was included in the model.

### Assessment of migrants' places (origin and destination) of initiating and continuing sex

Information on participants’ sexual history on timing of initiating sex at the place of origin (prior to first move, during current visit, between first move and current visit) and initiating sex at place of destination was used to describe the place of initiating and continuing sex with either paid or unpaid partners. Using this information, the descriptive statistics estimated (a) the proportion of men who continued to have sex in the place of destination, among those who initiated sex at the native place before their first move, and (b) the proportion of men who continued to have sex in the native place among those who initiated sex in the destination place. Additionally, the question on consistent condom use in sex with each type of partner (in order to reduce recall bias with condom use in a specific number of episodes) in each of the places of origin and destination during migration history was used to describe safe sex behavior in these places.

## Results

### Participant characteristics by migration status

The demographic characteristics of the participants by migration status for Prakasam and Azamgarh districts are shown in Table 1. In both the study districts, returned migrants were older than non-migrants and active migrants. Relatively higher proportions of returned migrants were currently married than active migrants and non-migrants. In Prakasam district, a relatively higher percentage of non-migrants than the migrants had completed high school (42 percent vs. 34 percent, z-value=2.60; p=0.009). Similar findings are noted in Azamgarh district. The large majority of surveyed men (irrespective of migration status) from Prakasam district were engaged in agricultural work, while fewer in Azamgarh district reported that they were engaged in agricultural work. The mean age at first migration for migrants in the study districts ranged between 18 and 20 years. Both migrants and non-migrants in Prakasam district had initiated sex at the age of about 19 years. In Azamgarh district, returned migrants had initiated sex about a year later than non-migrants or active migrants. In both districts, migrants had spent an average of 5-6 months at home during their last visit to their place of origin. Reasons for their last visit to the place of origin included agriculture purpose (Prakasam - 5 percent, Azamgarh - 22 percent ), vacation (Prakasam - 1 percent, Azamgarh - 27 percent ), to attend a marriage/function (Prakasam - 6 percent, Azamgarh - 15 percent ), for rest/break in between work (Prakasam - 84 percent, Azamgarh - 23 percent ), to attend a festival (Prakasam - 4 percent, Azamgarh - 9 percent).

**Table 1:** Socio-demographic characteristics of non-migrants, returned migrants and active migrants in Prakasam and Azamgarh districts, India

	Non-migrants	Returned migrants	Active migrants	Total
	
	%	%	%	
**Prakasam district**				
**Total sample size**	401	317	316	
*Age* (*Mean*±*SD*)	26.6 ± 6.1	28.8 ± 5.1	26.5 ± 5.5	27.3 ± 5.7
*Currently married*	66.8	86.4	70.6	74.0
*Median age at marriage*	20.0	21.0	20.0	20.0
*Education*				
*Illiterates*	10.5	8.2	10.8	9.9
*High school and above education*	42.4	30.3	38.3	37.4
Engaged in agricultural work	38.7	24.9	22.5	29.5
Age at first migration (Mean±SD)	--	20.3 ± 3.9	18.8 ± 3.9	19.6 ± 4.0^#^
Duration of stay in place of origin in the last visit*	--	-	5.7±4.6	-
Age at first sex (Mean±SD)	18.9±2.5	18.9±2.2	18.7±2.3	18.8±2.3

**Azamgarh**				
**Total sample size**	431	319	320	
Age (Mean±SD)	28.5 ± 6.7	35.2 ± 5.8	27.6 ± 6.1	30.2 ± 7.1
Currently married	67.8	96.9	71.3	77.5
Median age at marriage	19.5	20	20	20
Education				
*Illiterates*	7.2	3.1	5.3	5.4
*High school and above education*	39.2	34.2	51.9	41.5
Engaged in agricultural work	12.3	5.0	3.8	7.8
Age at first migration (Mean±SD)	--	19.1 ± 3.6	18.7 ± 3.2	18.9 ± 3.4^#^
Duration of stay in place of origin in the last visit	--	-	4.8±5.1	-
Age at first sex (Mean±SD)	18.4±3.6	19.6±4.0	17.5±3.8	18.5±3.9

^#^ Computed only for migrants.

* Computed for the last visit of migrants (active) to their native place.

### Association between migration status and risky sexual behavior

The proportion of men having sex with paid or casual unpaid partners in the past 12 months shows significant variation between non-migrants and migrants (Table 2). In both districts, active and returned migrants engaged more often than non-migrants in risky sexual behavior. For example, active migrants from Prakasam district were significantly more likely than non-migrants to report sex with a sex worker (14.6 percent vs. 7.5 percent; z-value= 3.06, p=0.002), sex with a casual unpaid partner (34.5 percent vs. 25.2 percent; z-value=2.72, p=0.007), sex with both a sex worker and a casual partner (13.9 percent vs. 7.5 percent; z-value=2.81, p=0.005) and sex with more than three sexual partners (17.7 percent vs. 9.2 percent; z-value=3.36, p<0.001) in the 12 months prior to the survey. Similarly, active migrants from Azamgarh district were significantly more likely than non-migrants to report sex with a sex worker (19.4 percent vs. 6.5 percent; z-value=30.53, p<0.001), sex with both a sex worker and unpaid casual partners (4.2 percent vs. 15.3 percent; z-value=32.60, p<0.001) and risky sexual behavior (6.8 percent vs. 16.5 percent; z=value=12.42, p=0.002) in the 12 months prior to the survey. Consistent condom use in sex with sex workers in both districts was higher than with casual unpaid partners.

**Table 2:** Number and type of sex partners, unprotected sex practices, STI risk of non-migrants, returned migrants and active migrants in Prakasam and Azamgarh districts, India

	Prakasam district				Azamgarh district			
	Non-migrants	Returned migrants	Active migrants	p-value^1^	Non-migrants	Retuned migrants	Active migrants	p-value^1^

**Total sample size**	**401**	**317**	**316**		**431**	**319**	**320**	

	**%**	**%**	**%**		**%**	**%**	**%**	

**Ever had sex with**								
Either sex worker or casual unpaid partner	40.4	57.7	57.6	<0.001	38.3	42.3	47.8	0.033
Male partner	1.5	0.9	1.9	0.602	3.0	4.4	1.9	0.184
**Had sex in past 12 months with**								
Sex worker	7.5	12.0	14.6	0.009	6.5	10.3	19.4	<0.001
Casual unpaid partner	25.2	33.4	34.5	0.011	21.1	16.9	27.5	0.005
Both sex worker and casual partner	7.5	11.4	13.9	0.019	4.2	6.3	15.3	<0.001
3+ sex partners	9.2	15.5	17.7	0.003	10.4	10.0	17.2	0.007
**Consistent condom use^#^ in past 12 months with**								
Sex worker^2^	76.7 (30)	68.4 (38)	73.9 (46)	0.732	28.6 (28)	42.4 (33)	41.9 (62)	0.435
Casual unpaid partner^4^	15.8 (101)	22.6 (106)	31.2 (109)	0.031	11.0 (91)	16.7 (54)	33.0 (88)	0.001
**Risky sexual behavior^$^**	9.3	12.3	13.1	0.334	6.8	8.6	16.5	0.002

^1^ Chi-square test.

^2 ^Among those who had sex with sex workers in last 12 months.

^3^ Among those who had sex with sex workers.

^4 ^Among those who had sex with casual unpaid female partners in last 12 months.

^5^ Among those who had sex with casual unpaid female partners.

^#^ Every time condom use in sex episodes in past12 months prior to the survey with specific type of partner (irrespective of place of origin or destination).

( ) Data in parenthesis indicates the total sample size for that variable.

^$^ No consistent condom use in sex with both sex worker and/or casual unpaid partner, and having sex with both sex worker and casual unpaid partners.

Table 3 presents the results of multiple logistic regression analyses, which confirm the differences noted above in risky sexual behavior between migrant and non-migrant men. The results show that even after controlling for socio-demographic characteristics, risky sexual behaviors are higher among both active and returned migrants than non-migrants. Returned migrants were two times more likely to have had sex with paid partners in last 12 months (in Prakasam: aOR=1.7, 95% CI: 1.0-2.9; in Azamgarh: aOR=1.7, 95% CI: 1.0-2.9) than non-migrants. Similarly, active migrants were significantly more likely to have sex with paid partners than non-migrants (in Prakasam: aOR=2.1, 95% CI: 1.2-3.4; in Azamgarh: aOR=4.0, 95% CI: 2.4-6.6). The likelihood of having sex with a casual unpaid partner is significantly higher among migrants than non-migrants in Prakasam district but not in Azamgarh district. Consistent condom use with a casual unpaid partner was reported to be higher among active migrants than non-migrants in both the study districts (in Prakasam: 31 percent vs. 16 percent, aOR=2.3, 95% CI: 1.1-4.6; in Azamgarh: 33 percent vs. 11 percent, aOR=4.0, 95% CI: 1.6-9.9).

**Table 3:** Associations between migration status and sexual behaviors, unprotected practices of study participants in Prakasam and Azamgarh districts, India

	Prakasam district		Azamgarh	
	
	Returned migrants/Non-migrants	Active migrants/Non-migrants	Returned migrants/Non-migrants	Active migrants/Non-migrants
	
	AOR^1^ (95% CI)	AOR^1^ (95% CI)	AOR^1^ (95% CI)	AOR^1^ (95% CI)
	
**Ever had sex with**				
Either sex worker or casual unpaid partner	1.85 (1.36-2.54)*	1.86 (1.36-2.53)*	1.19 (0.86-1.64)	1.43 (1.06-1.96)**
Male partner	0.56 (0.13 – 2.40)	1.19 (0.36 – 3.89)	1.78 (0.73 – 4.31)	0.60 (0.22 – 1.64)
**Had sex in past 12 months with**				
Sex worker	1.72 (1.02 – 2.91)**	2.05 (1.24 – 3.38)*	1.68 (0.96 – 2.94)	4.00 (2.43 – 6.59)*
Casual unpaid partner	1.57 (1.11 – 2.23)**	1.50 (1.06 – 2.11)**	0.95 (0.63 – 1.43)	1.41 (0.98 – 2.02)
Both sex worker and casual partner	1.63 (0.95 – 2.77)	1.93 (1.17 – 3.20)**	1.44 (0.72 – 2.88)	4.45 (2.47 – 8.02)*
3+ sex partners	1.78 (1.10 – 2.88)**	2.03 (1.28 – 3.22)*	1.22 (0.72 – 2.06)	1.92 (1.23 – 3.01)*
**Consistent condom use^#^ in past 12 months with**				
Sex worker^2^	0.98 (0.29-3.31)	0.80 (0.25-2.60)	1.42 (0.46-4.42)	1.53 (0.55 - 4.23)
Casual unpaid partner^4^	1.40 (0.66-3.00)	2.29 (1.13-4.60)**	1.15 (0.39-3.39)	4.00 (1.62 - 9.93)*
**Risky sexual behavior^$^**	1.79 (1.01-3.19)*	1.54 (0.87-2.72)	1.46 (0.68-3.12)	3.05 (1.59-5.87)

AOR, Adjusted odds ratio; CI, confidence interval; STI, sexually transmitted infection.

The independent variable coding for the odds ratios presented are: Returned migrants (coded as 1) /Non-migrants (coded as 0); Active migrants (coded as 1) /Non-migrants (coded as 0).

^1^ Controlled for age, education, marital status and occupation.

^2^ Among those who had sex with sex workers in last 12 months.

^3^ Among those who had sex with sex workers.

^4^ Among those who had sex with casual unpaid female partners in last 12 months.

^5^ Among those who had sex with casual unpaid female partners.

^#^ Condom use at last time sex in 12 months prior to the survey with specific type of partner (irrespective of place of origin or destination).

* p<0.01, ** p<0.05.

### Where do migrants initiate risky sexual behavior?

Results presented in Table 4 indicate that sexual relationships among migrants initiated at the place of origin were primarily non-commercial. A higher proportion of migrants initiated sex with a paid partner at the place of destination than at the place of origin. However, a higher proportion of migrants initiated sex with a casual unpaid partner at the place of origin than at the place of destination.

**Table 4:** Migrants’ initiation into sex work and their continuation of sex between places of origin and destination

	Prakasam district			Azamgarh district		
	Sex worker	Casual unpaid partner	Any partner^#^	Sex worker	Casual unpaid partner	Any partner^#^

	%	%	%	%	%	%

**Total sample size**	**633**	**633**	**633**	**639**	**639**	**639**
Migrants who never had sex	75.0	44.9	42.0	75.9	54.1	48.8
Migrants who initiated sex in place of origin	9.8	42.5	43.4	7.5	42.7	43.7
Migrants who initiated sex in place of destination	15.2	12.6	14.5	16.6	3.1	7.5
**p-value**	**<0.001**	**<0.001**	**<0.001**	**<0.001**	**<0.001**	**<0.001**
**Number who initiated sex in place of origin**	**62**	**269**	**275**	**48**	**273**	**279**
Had sex in destination after initiating at place of origin [total]	59.7	39.4	46.9	49.0	23.5	47.7
*Had sex in destination only*	19.4	6.7	6.9	29.2	2.6	9.0
*Had sex both in destination and in origin during their migration*	40.3	32.7	40.0	18.8	20.9	38.7
Had sex in only origin areas during their migration	16.1	39.4	32.7	43.8	53.1	35.5
Did not have sex in any area during migration after they first had sex before migration	24.2	21.2	20.4	8.3	23.4	16.9
**Number who initiated sex in place of destination**	**96**	**80**	**92**	**106**	**20**	**48**
Had sex in destination areas only but not in origin	45.8	56.3	54.4	85.9	75.0	79.2
Had sex in both destination and origin areas	54.2	43.8	45.7	14.2	25.0	20.8

^#^ Sex either with sex worker and/or casual unpaid female partner.

Among those who initiated sex with a female partner (either a sex worker or a casual unpaid partner) in the place of origin, nearly half (47 percent in Prakasam and 48 percent in Azamgarh) in both the districts reported continuing the practice at the place of destination. Surprisingly, a considerable proportion of those who initiated sex in the place of origin (Prakasam: 33 percent, Azamgarh: 36 percent) reported having sex only at the place of origin during migration and not at the destination area. Nearly half of the migrants (46 percent) in Prakasam and one-fifth (21 percent) in Azamgarh who had initiated non-spousal sex at the place of destination continued the practice at the place of origin as well. Results in Table 5 suggest that among migrants, consistent condom use in sex with either a sex worker or a casual unpaid partner is lower in the place of origin than in the place of destination. For instance, in Azamgarh district, only 14 percent (n/N=3/21) of returned migrants who had sex with sex workers in place of origin in present visit reported consistent condom use as compared to 54 percent of those returned migrants who had sex with sex workers in the places of destination during migration reported consistent condom use (p<0.05). Similar results are noted for returned migrants on condom use in sex with either sex workers or casual unpaid partners in Prakasam district. The consistent condom use in sex with either sex workers or casual unpaid partners by active migrants in Prakasam district (as a place of origin) is significantly low as compared to level of consistent condom use by these men in sex at the places of destination.

**Table 5:** Percent male migrants who reported consistent condom use^$^ during sex in places of destination and places of origin by type of partner

Consistent condom use in sex with	Prakasam district			Azamgarh district		
	
	Sex worker	Casual unpaid partner	Any partner^#^	Sex worker	Casual unpaid partner	Any partner^#^
	
	% (n/N)	% (n/N)	% (n/N)	% (n/N)	% (n/N)	% (n/N)
**Both returned and active migrants sample^@^**	**158**	**349**	**367**	**154**	**293**	**327**
At place of origin before first move	72.4 (42/58)	19.6 (43/220)*	28.9** (68/235)	36.4 (12/33)	17.2* (44/256)	19.1** (50/262)
At place of origin during migration	59.4* (41/69)	25.9 (45/174)	38.8** (76/196)	30.6* (11/36)	27.9 (53/190)	28.1** (57/203)
At place of origin in present visit	71.9 (41/57)	20.0 (38/190)	35.9** (71/198)	23.3** (7/30)	26.1 (36/138)	27.2** (40/147)
At places of destination during migration^(R)^	74.4 (99/133)	26.9 (50/186)	55.7 (123/221)	53.5 (69/129)	27.4 (23/84)	44.2 (80/181)
**Returned migrants sample^@^**	**77**	**176**	**182**	**84**	**142**	**159**
At place of origin before first move	64.0 (16/25)	21.2 (24/113)	28.9** (35/121)	25.0** (4/16)	13.1** (16/122)	14.3** (18/126)
At places of origin during migration	67.7 (23/34)	27.0* (24/89)	40.4 (40/99)	23.8* (5/21)	25.0 (23/92)	24.8** (25/101)
At places of origin in present visit	76.7 (23/30)	19.8 (19/96)	38.0* (38/100)	14.3** (3/21)	24.2 (16/66)	25.4** (18/71)
At places of destination during migration^(R)^	71.2 (47/66)	22.7 (20/88)	51.8 (57/110)	54.1 (40/74)	31.6 (12/38)	46.9 (45/96)
**Active migrants sample^@^**	**81**	**173**	**185**	**70**	**151**	**168**
At place of origin before first move	78.8 (26/33)	17.8* (19/107)	29.0** (33/114)	47.1 (8/17)	20.9 (28/134)	23.5** (32/136)
At place of origin during migration	51.4** (18/35)	24.7 (21/85)	37.1** (36/97)	40.0 (6/15)	30.6 (30/98)	31.4 (32/102)
At place of origin in present visit	66.7 (18/27)	20.2 (19/94)	33.7** (33/98)	44.4 (4/9)	27.8 (20/72)	29.0 (22/76)
At places of destination during migration^(R)^	77.6 (52/67)	30.6 (30/98)	59.5 (66/111)	52.7 (29/55)	23.9 (11/46)	41.2 (35/85)

n - number used condoms consistently in sex; N - number who had sex with that specific partner within that place.

^@^ - Total sample who had sex with the specific partner either in place of origin or place of destination.

* p<0.05, ** p<0.01

^(R) -^ Reference Category for comparison between proportions.

^$^ - Based on separate questions that asked about every time condom use in sex with different type of partners (sex worker or casual unpaid female partner) in places of origin and destination.

^#^ - Consistent condom use in sex either with sex worker or casual unpaid or both partners (as applicable) in places of origin and/or destination.

Note: p-value refers to test for difference between proportions. Place of destination is used as the reference category to measure the differences in condom use behavior at place of origin at different times during the migration.

## Discussion

The current study documents higher rates of risky sexual behaviors among migrant than non-migrant men in the place of origin, a finding consistent with previous research studies conducted in the place of destination [1,20-24,38]. Our study results also suggest that returned migrants continue to practice such behaviors in the place of origin and a higher proportion of active migrants are engaged in risky sexual behaviors in the place of origin than in destination areas. Further, results suggest that initiation of sexual behavior among migrants is mostly connected to their native place rather than their destination place. Most migrants initiate sex in the place of origin before their first migration to a destination area, and continue to practice such activities in the place of destination. However, migrants’ behaviors in destination areas are strongly linked to their exposure to sex workers, and their behaviors in the place of origin are strongly linked to sex with casual unpaid female partners. These findings are consistent with growing evidence from India over the past few years documenting a growing concern regarding the sexual behavior of individuals and increasing HIV incidence among ANC clinic attendees in places of origin that are known to have very low HIV prevalence a decade ago [31,39,40]. More importantly, a considerable proportion of non-migrant men were also found to have engaged in sex with sex workers as well as casual unpaid partners, which indicates the existence of a high level of local sexual networks in the places of origin. This, in combination with unprotected sex among non-migrant and returned migrant men in places of origin and unprotected sex with sex workers among migrants in destination areas, may contribute to the spread of the HIV epidemic in the places of origin.

Notably, few migrants reported sexual debut with a casual unpaid partner in the destination areas. Those who initiated sexual activity with a paid or unpaid partner in the destination area continued to have sex with such partners upon return to the place of origin, particularly in Prakasam district. The pattern of sexual behavior in Prakasam district (largely within-state migration) seems to parallel the situation in other settings, which indicates that the enhanced social status of migrants and exposure to commercial sex in the destination place enables them to seek and receive sexual favors more successfully from women in their village of origin [41]. Perhaps, in the case of Prakasam district, the high frequency of migration to the place of origin between periods of work in the destination area provides them more opportunities to seek and practice the sexual risk behaviors that were initiated in places of destination. In contrast, in Azamgarh district, the large majority of those who initiated sex in the destination area continued to have sex only in the destination area and not in the place of origin. Unprotected sex by migrants is relatively higher in places of origin than in places of destination, and more so in Azamgarh district than in Prakasam district. Relatively high rates of consistent condom use by migrants (particularly with sex workers) in Prakasam district of Andhra Pradesh than the Azamgarh district of Uttar Pradesh can be explained by the differences in intensive HIV prevention efforts in Andhra Pradesh state versus Uttar Pradesh state [40].

Nonetheless, unprotected sex is high in places of origin in general, though patterns differ for migrants and non-migrants. Although the percentage of migrants having sex with sex workers in the place of origin is relatively low, almost all those who had sex with paid partners also had sex with a casual unpaid partner, a finding that highlights the concurrent relationships in this group of men. Moreover, unprotected sex with casual unpaid sexual partners among non-migrant as well as migrant men was much higher than unprotected sex with paid partners. This may be because casual unpaid partners are known and trusted individuals living within their area or nearby. An exploratory analysis in this direction from the study data in Prakasam district indicates that about one-third of men who had sex with casual unpaid partners reported their last sexual partner was a neighbor while about one-fourth reported that their last sexual partner was a friend or acquaintance. In Azamgarh district, about half of the total men who had sex with a casual unpaid partner reported that their last sexual partner was a neighbor, and one-fifth reported that their last sexual partner was a relative. These findings suggest that infected returned or active migrants are likely to transmit infection to their neighbors, friends, and relatives in places of origin through the continuation of risky sexual behavior.

In sum, the overall level of risky sexual behaviors varies significantly by district, as do the associations between migration and unprotected sex with different type of partners. There could be several reasons for such differences. First, men in Prakasam district migrate mainly to neighboring districts (short distance migration) within the state of Andhra Pradesh whereas men from Azamgarh district migrate mostly to other states (long distance migration) particularly to the state of Maharashtra. Such short distance migration provides them opportunity for frequent visits to the native place where they may have already established sexual networks with other women. The availability of disposable money and frequent visits create opportunities for expanding existing sexual networks at the native place. In contrast, migrants from Azamgarh district visit their native place less frequently and therefore probably have less opportunity to continue or expand their sexual networks at their native place. Second reason for such variations in the level of sexual risk behaviors may be that in contrast to migrants from Azamgarh, those from Prakasam do not face language or cultural barriers as their place of destination and origin are similar. The lack of cultural barriers is an advantage for migrants when buying sex from sex workers or soliciting sex from casual unpaid partners in both the place of destination and origin. This can be substantiated by the finding that a higher proportion of multiple sexual partners are reported in Prakasam district than in Azamgarh district. In contrast, a small proportion of migrants from Azamgarh district initiated sex with a casual unpaid partner at the place of destination. Moreover, the proportion of migrants who initiated non-spousal sex at the place of origin and continued similar practices at the place of destination is quite low. This result to an extent supports the finding that language barriers faced by the migrants in case of inter-state migration play a critical role in limiting risky behaviors at the places of destination [24]. Third, these differences may also reflect cultural variations to the extent that a much higher proportion of men in south India have non-regular sexual partners than in the north [34]. The persistent risk of HIV transmission is notable given that both the districts included in the study show high and rising HIV prevalence among women attending ANC clinics [34]. For women attending ANC clinics, the primary source of infection may be sex with infected men—migrant husband as well as others when husband is away. Due to lack of information on sexual networks of women in the place of origin as well lack of biological data in the current study, it was difficult to document the potential role of migration in the spread of HIV infection to both men and women and to their married partners. Further research is needed to understand the migration-related spread of HIV infection versus the spread through local sexual networks in districts with high out-migration and high HIV prevalence. Research is also needed to examine systems of sexual networks for both men and women and the potential utility of tapping such networks for HIV prevention interventions in selected places of origin. Some insights into this issue are provided via an exploratory analysis in the current study, which indicates that a large proportion of non-migrants and migrants reported having sex with sex workers as well as casual unpaid partners including unmarried women, married women in the neighborhood, as well as wives of migrant men who have been left behind. These results highlight that there are specific groups of sexual partners with whom these different categories of migrant men continue to have sex in the place of origin. In the context of rural India (often the places of origin), there are complex sexual relationships men may have with women that pose greater risk for HIV, particularly migrant men’s wives who have been left behind.

Findings from this study have important implications for the current strategies and design of national HIV/AIDS prevention programs. The existing intervention approaches in places of destination [1] among migrants completely ignore the fact that many migrants initiate sex outside marriage and some initiate sex with sex workers even in their native place. Existing interventions in destination areas are based on the assumption that condom use promotion among female sex workers can protect migrant men and thus prevent further transmission, but do not consider secondary transmission from infected migrant men to their spouses and other female populations, including sex workers in the places of origin. More importantly, interventions in the destination areas do not focus on returned migrants as well as on non- migrants who represents a pool of potential migrants. The present study shows that a greater proportion of returned migrants continue to have unprotected sex with sexual partners in their native place and a considerable proportion of non-migrants also engage in risky sexual behavior. Moreover, both groups initiated sex at about the same age. These results indicate the need for initiating HIV prevention efforts in places of origin with high out-migration. While such efforts may not be necessary in all places of origin because such places are difficult to identify, initial efforts could focus on districts identified to have high male out-migration and emerging HIV prevalence among women in ANC clinics or men in integrated counseling and testing centres (ICTCs). Through this strategy, potential migrants can be counseled about safe sex practices even before they migrate as well as informed about HIV prevention services available at places of destination.

While the present study contributes important information to guide future research and programmatic work relevant to primary and secondary HIV prevention in districts with high out-migration in India, it must be considered in the light of a few study limitations. The sample was drawn from two high HIV prevalence districts with net out-migration at the district level, and included a sample of men from households in the villages who were available at the time of survey. These factors may limit the generalization of findings to a total sample of migrants and non-migrants in districts with high out-migration. However, given that the data were collected from areas with within-state and out-of-state migration, it is expected that the potential risks in patterns of migration would be reduced while examining the linkage between migration and HIV risk behaviors of men in their native place. Additionally, much of the data in this study came from self-reports of behavior and covered the period of their migration history, and are thus subject to both social desirability and recall biases, particularly in case of retrospective data from migrant men. Male interviewers of similar age were recruited to increase comfort and reduce social desirability bias. Additionally, major events were referred in the questionnaire, such as prior to first move, during migration at the destination areas, during migration at the place of origin and in the current visit, to reduce recall biases in retrospective data. Finally, the use of retrospective information rather than the prospective longitudinal data precludes claims of causality based on the observed associations. Additional research with longitudinal data would be useful to confirm current findings and explore in greater detail issues that could not be explained from the results.

## Conclusions

The present study contributes to the growing literature on sexual risk among populations in their place of origin in India by examining risky sexual behavior among three categories of men - active migrants, returned migrants and non-migrants. Findings document that a higher proportion of both returned and active migrants engaged in risky sexual behaviors than the non-migrants. Most migrants initiate non-marital sex in the place of origin and many continue these behaviors in places of destination. Migrants’ destination area behaviors are linked to sex with sex workers and they continue to practice such behaviors in the place of origin as well. Unprotected sex in places of destination with high HIV prevalence settings poses a risk of transmission from high risk population groups to migrants, and in turn to their married and other sexual partners in native places. More importantly, the finding that returned migrants reported having sex with unpaid sexual partners, such as unmarried women or wives of married migrant men left behind, points to an area of concern in places of origin. These findings support the need for HIV prevention interventions in selected places of origin with high male out-migration and emerging HIV prevalence among ANC clinic attendees or ICTC centres in India. These interventions would need to be tailored to focus on the populations most at risk for HIV – potential and active migrants and their sexual partners including left-behind wives.

## List of abbreviations used

AIDS: Acquired immune deficiency syndrome; AOR: Adjusted Odds Ratio; CI: Confidence Interval; HIV: Human Immunodeficiency Virus; NACO: National AIDS Control Organisation; NGO: Non-Governmental Organization; ANC: Antenatal care; PHC: Primary Health Centre; ICTC: Integrated Counseling and Testing Centre; SPSS: Statistical Package for Social Sciences; STI: Sexually Transmitted Infections.

## Competing interests

The authors have no financial benefits or competing interests related to this submitted work.

## Authors’ contributions

NS led the study design, conception, analyses, and drafted the manuscript. BM assisted with the analyses and manuscript writing. SNS participated in the design of the study and performed the statistical analysis. AKJ provided overall guidance with analytical approach and interpretation of study findings. All authors read and approved the final manuscript.
